# Heat Priming During Early Reproductive Stages Enhances Thermo-Tolerance to Post-anthesis Heat Stress via Improving Photosynthesis and Plant Productivity in Winter Wheat (*Triticum aestivum* L.)

**DOI:** 10.3389/fpls.2018.00805

**Published:** 2018-06-13

**Authors:** Yonghui Fan, Chuanxi Ma, Zhenglai Huang, Muhammad Abid, Suyu Jiang, Tingbo Dai, Wenjing Zhang, Shangyu Ma, Dongguo Jiang, Xiao Han

**Affiliations:** ^1^Key Laboratory of Wheat Biology and Genetic Improvement on South Yellow and Huai River Valley of China, Ministry of Agriculture, Anhui Agricultural University, Hefei, China; ^2^Department of Soil and Water Conservation, Khushab, Pakistan; ^3^Key Laboratory of Crop Physiology, Ecology and Production Management, Nanjing Agricultural University, Nanjing, China

**Keywords:** heat priming, thermo-tolerance, photosynthesis, grain yield, winter wheat (*Triticum aestivum* L.)

## Abstract

Heat stress during grain filling substantially decreases wheat productivity; thus, to ensure food security, heat tolerance in wheat needs to be developed. In this study, we evaluated the effect of heat priming applied during the stem-elongation stage, booting and anthesis, followed by 5 days of severe heat stress (a 7.86°C rise in temperature) during the grain-filling stage on physiological activities and grain yield of winter wheat in pot experiments during the 2015-2017 growing seasons using the winter wheat cultivars Yangmai 18 (a vernal type) and Yannong 19 (a facultative type). Compared with the damage observed in non-primed plants, heat priming during the stem-elongation stage and booting significantly prevented the grain-yield damage caused by heat stress during grain filling. Heat-primed plants displayed higher sucrose contents and sucrose-phosphate activity in leaves and greater above-ground dry matter than non-primed plants. Priming during stem elongation and booting led to increased photosynthetic capacity, stomatal conductance and chlorophyll contents in comparison with non-priming. Improved tolerance to heat stress due to the enhanced activities of antioxidant enzymes superoxide dismutase and peroxidase and reductions in reactive oxygen species and malondialdehyde production was observed in primed plants compared with non-primed plants of both cultivars. The positive effect of heat priming on the response to heat stress during grain filling was more pronounced in plants primed at the booting stage than in those primed at the stem-elongation or anthesis stage. Moreover, the vernal-type Yangmai 18 benefited more from heat priming than did Yannong 19, as evidenced by its higher productivity. We conclude that heat priming during early reproductive-stage growth can improve post-anthesis heat tolerance in winter wheat.

## Introduction

Atmospheric temperatures have increased since the beginning of the 21st century and are predicted to continue increasing, with the global mean air temperature predicted to increase by approximately 1.0–1.7°C by 2050 (Intergovernmental Panel on Climate Change [IPCC], 2014). Increases in atmospheric temperature and in the frequency of extreme weather may expose crops to multiple extreme heat events during the growing season (Lobell et al., 2011). Thus, the response of plants to high temperature events at different growth stages may have important implications for the development of stress tolerance in crops. Heat stress severely affects plant growth and development and is classified as a major abiotic stressor for numerous crops (Moriondo et al., 2011).

Wheat (*Triticum aestivum* L.) is a widely grown cereal crop that provides food for more than 35% of the global population. Wheat is grown on more than 200 million hectares worldwide, and more than 600 million tons are produced annually (Food and Agriculture Organization [FAO], 2013). However, the productivity of wheat is severely affected by heat stress (Lobell et al., 2012). Average optimal temperatures for wheat growth range from 17 to 23°C (Porter and Gawith, 2015), and when the temperature exceeds this range, the plant is considered to be under heat stress, a condition that may cause irreversible damage to crop growth and development. Heat stress can affect wheat growth and productivity at all stages of growth, and if heat stress occurs during the reproductive phase, substantial yield losses are incurred because of the direct effect of heat stress on grain number and mass (Talukder et al., 2014).

Heat stress affects plant growth and development through physiological injury to membrane lipids, carbon and nitrogen metabolism, photosynthesis, yield formation, and grain quality (Asseng et al., 2011). Photosynthesis is highly sensitive to temperature. Heat stress decreases photosynthesis by disrupting chloroplast structure and function, thereby reducing the chlorophyll content and accelerating the loss of green leaf area, which is negatively associated with grain yield in wheat (Dias et al., 2011). During early reproductive stages, efficient photosynthesis and photosynthate partitioning play a decisive role in dry matter (DM) assimilation and in the formation of reproductive organs (Masoni et al., 2007). Sharkey (2005) reported that under heat stress, the limited supply of photosynthate may reduce grain filling in wheat due to reduced activity of key enzymes involved in starch accumulation in the grains. In addition, Jenner (1994) indicated that in wheat, a short period of episodic temperatures above 30°C slows starch accumulation and reduces grain growth because heat stress induces denaturation of the soluble enzyme starch synthase. The effects of high temperature on photosynthesis are also associated with impaired photochemical reactions in thylakoids and carbon assimilation reactions in the chloroplast stroma (Ristic et al., 2007). Moreover, heat stress results in oxidative stress associated with the production of reactive oxygen species (ROS), ultimately affecting the structure of thylakoid membranes, photosystem II (PSII) activity, and chlorophyll (Sharkey, 2005). Many studies have indicated that ROS scavenging plays a significant role in protecting plants from heat stress. Thus, plant tolerance to abiotic stress is closely related to the capacity to scavenge and detoxify ROS, which largely occurs through the activity of such antioxidant enzymes as superoxide dismutase (SOD) and peroxidase (POD) (Wang et al., 2014).

Projected climatic and environmental variations highlight the need for developing strategies that promote both substantial increases in yield potential and resilience to extreme heat events, especially during the grain-filling phase of wheat growth. Plants able to grow and produce economic yields under heat stress are called thermo-tolerant (Wahid et al., 2007). To date, attention has predominantly focused on priming induced by exogenously applied chemicals (Farooq et al., 2010; Li et al., 2011; Zahoor et al., 2017), though a few studies have investigated abiotic stress-induced priming techniques (Xu et al., 2006; Talukder et al., 2013; Li et al., 2014a). Some recent reports have shown that pre-exposure priming of plants to stress conditions can promote tolerance to stress conditions that occur during subsequent growth periods (Walter et al., 2011; Backhaus et al., 2014). Our previous studies revealed that drought priming during early growth stages of winter wheat facilitates the maintenance of plant growth and grain development during post-anthesis drought conditions by modulating plant physiological processes (Wang et al., 2014; Abid et al., 2016). Zhang et al. (2016) reported that thermo-tolerance induced in wheat plants by heat priming can even be ‘remembered’ and inherited by offspring. Yucel et al. (1992) reported that a 37°C heat-stress treatment for 30 min decreased the photosynthetic capacity of wheat but that photosynthetic capacity completely recovered after relieving the heat treatment in pre-heat treated seedlings. Similarly, Wang et al. (2014) indicated that multiple heat priming of wheat seedlings increased winter wheat thermo-tolerance to a later heat stress by enhancing subcellular antioxidant activities; nevertheless, the time between priming and the subsequent stress was very short (several hours or days). Regardless, whether wheat plants can retain a ‘memory’ of a previous high-temperature stress episode during a subsequent heat-stress event, as well as the possible underlying mechanisms, remains largely unknown. In addition, limited information is available about photosynthetic characteristics, ROS generation and antioxidative system performance in heat-primed and non-primed plants during subsequent heat stress.

The main goal of this paper was to examine whether heat priming applied during the stem-elongation stage, booting or anthesis can induce thermo-tolerance to heat stress during filling in winter wheat. The results of the present study are expected to support research efforts seeking to develop heat-stress tolerance in winter wheat.

## Materials and Methods

### Experimental Site

A 2-year pot experiment was conducted from 2015 to 2017 at Nongcuiyuan Experimental Research Station of Anhui Agriculture University, Hefei (32°04′N, 118°76′E), Anhui Province, China. Rainfall and average daily temperature from sowing to maturity were 521.5 mm and 11.3°C in 2015-2016 and 542.8 mm and 12.0°C in 2016-2017, respectively.

### Experimental Design

Wheat plants were grown outdoors in 25-cm diameter, 30-cm high pots according to Li et al. (2014b), Wang et al. (2014), Abid et al. (2016), and Zhang et al. (2016). Two local cultivars, vernal type Yangmai 18 and facultative type Yannong 19, were used. These two wheat cultivars show good productivity and adaptability in this area, and the optimal temperature of the vernal type during the vernalization phase is higher than that of the facultative type (Yang et al., 2014; Mu et al., 2015). The sowing dates during the two experimental years were November 8, 2015, and November 4, 2016. Each pot was filled with 12.0 kg of clay soil containing 1.1 g kg^-1^ total N, 72.4 mg kg^-1^ available N, 21.9 mg kg^-1^ Olsen-P, 146.2 mg kg^-1^ available K, and 14.0 g kg^-1^ organic matter. Fertilizers (0.9 g N, 0.36 g P_2_O_5_, and 0.9 g K_2_O per pot) were applied before seeding and were completely mixed with the soil; an additional 0.3 g of N per pot was applied at the stem-elongation stage (Zadoks growth Stage 31) (Zadoks et al., 1974). The plants were thinned to eight seedlings per pot at the three-leaf stage (Zadoks growth Stage 13). Pesticides and fungicides were applied at the stem-elongation (Zadoks growth stage 31) and booting (Zadoks growth stage 41) stages to protect against pests and diseases.

Heat priming treatments were applied by moving the pots to a 4.5 m × 4.5 m × 3.5 m (L × W × H) glass chamber according to Mohammed and Tarpley (2010), Shi et al. (2013), and Talukder et al. (2014), with minor modifications. The glass chamber allowed approximately 90% of visible wavelengths, with high solar transmittance. The effect of reduced UV radiation (90% UV transmittance) can be ignored. The chamber was equipped with an electric heater (3000 W, CC-107, Ningbo Southeast Co., China) and an air conditioner (1500 W, KFR-35GW, Zhuhai Gree Electric Appliances Co. China) capable of increasing the temperature uniformly throughout the chamber. We installed two inlets and two outlet fans (350 W, AH9, Ningbo Southeast Co., China) at the front and back wall of the chamber, with the aim of maintaining constant but gentle air exchange and minimizing differences in the relative humidity and CO_2_ concentration within the chamber compared with the ambient control. Relative humidity in the chamber was maintained at 60–70% by a humidifier (100 W, HU4902/00, Royal Dutch Philips Electronics Ltd., Holland). Each time we moved the pots into the heated glass chamber, the temperature inside the chamber was stable and uniform.

Five-day heat priming treatments (average temperature ∼5°C above the ambient control) were conducted at the stem-elongation (Zadoks growth stage 31; the 1st node was detectable), booting (Zadoks growth stage 41; flag leaf sheath-extending stage) and anthesis (Zadoks growth stage 60; i.e., the beginning of pollination) stages by moving the pots into the heated glass chamber. The remaining pots were moved to another glass chamber at ambient temperature. After heat priming at the different stages, all pots were subjected to field conditions. At 15 days after anthesis (DAA), all the temperature-primed pots and half of the control pots were subjected to heat stress for 5 days (the average temperature was ∼8.0°C above the ambient temperature) during the grain-filling stage (the Zadoks growth stage 73, early milk). At the same time, the remaining pots were moved to another glass chamber at ambient temperature. After the heat-stress treatment, all the pots were moved from the glass chambers and grown under field conditions. Plants were carefully noted to have reached a particular growth stage when more than 50% of the wheat plants reached that stage; the stages were determined according to Zadoks et al. (1974). The phenophase of the two wheat cultivars is provided in **Table 1**. Plants of each cultivar were treated when they reached their respective stage.

**Table 1:** Phenophase of the two wheat cultivars from 2015 to 2017.

Year	Cultivar	Phenophase date (day/month)				
		Sowing	Stem-elongation	Booting	Anthesis	Maturity
2015-2016	Yangmai 18	8th November	17th February	18th March	7th April	13th May
	Yannong 19	8th November	24th February	24th March	11th April	17th May
2016-2017	Yangmai 18	4th November	23rd February	26th March	14th April	21st May
	Yannong 19	4th November	31st February	2nd April	19th April	24th May

**FIGURE 1:**
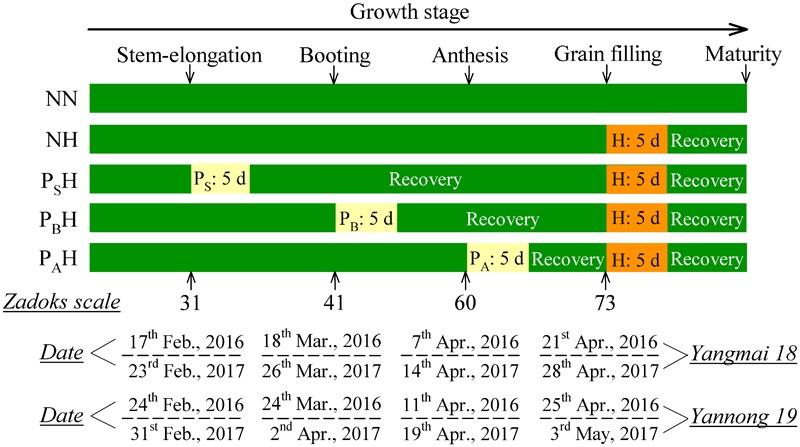
Schematic representation of the experimental design and treatments. P_S_, P_B_, and P_A_ refer to 5 days of heat priming at the stem-elongation, booting and anthesis stages, respectively. H refers to 5 days of heat stress at the grain-filling stage. NN refers to no heat priming + no heat stress at the grain-filling stage (control); NH refers to no heat priming + heat stress at the grain-filling stage; P_S_H refers to heat priming at the stem-elongation stage + heat stress at the grain-filling stage; P_B_H refers to heat priming at booting + heat stress at the grain-filling stage; P_A_H refers to heat priming at anthesis + heat stress at the grain-filling stage. The morphology date is indicated according to the Zadoks scale: Stage 31, the 1st node was detectable (stem-elongation stage); Stage 41, extension of the flag leaf sheath (booting); Stage 60, the beginning of pollination (anthesis); Stage 73, early milk (grain-filling stage).

**FIGURE 2:**
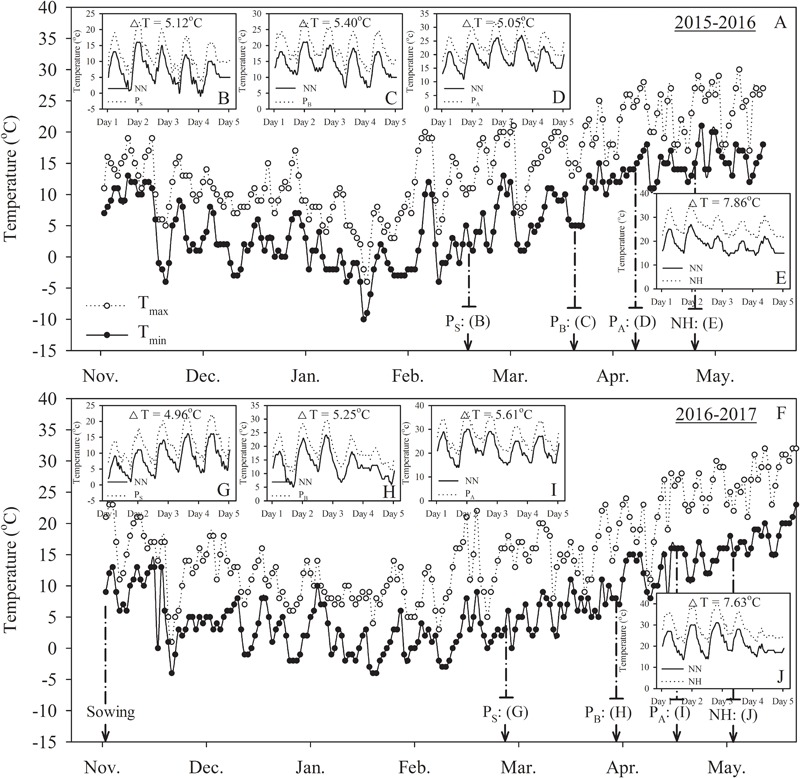
Changes in daily minimum and maximum temperatures for the crop canopy from sowing to maturity, the mean air temperature of the wheat canopy in the high-temperature treatments and normal temperature control during the 2015–2016 **(A–E)** and 2016–2017 **(F–J)** wheat-growing seasons. P_S_, P_B_, and P_A_ refer to 5 days of heat priming at the stem-elongation, booting and anthesis stages, respectively. NN refers to no heat priming + no heat stress at the grain-filling stage (control). NH refers to no heat priming + heat stress at the grain-filling stage. ΔT refers to the increase in mean temperature between the treatment and control (average of the two wheat cultivars). Mean temperature represents the mean of all temperature data collected at 10-min intervals during the treatments.

Five treatments were established: no heat priming + no heat stress at the grain-filling stage as the ambient temperature control (NN); no heat priming + heat stress at the grain-filling stage (NH); heat priming at stem-elongation stage + heat stress at the grain-filling stage (P_S_H); heat priming at booting + heat stress at the grain-filling stage (P_B_H); and heat priming at anthesis + heat stress at the grain-filling stage (P_A_H). The experimental design is shown in **Figure 1**. The temperature and relative humidity inside the chambers were automatically recorded every 10 min using a dual-channel LCD temperature instrument (RC-4HC, Shanghai Jingchuang Electronic Instrument Co., China) placed at canopy height. The average canopy temperature of the primed pots was 5.12°C (P_S_), 5.40°C (P_B_), 5.05°C (P_A_) and 7.86°C (H) higher than the control (NN) in the 2015-2016 season; in the 2016-2017 season, the values were 4.96°C (P_S_H), 5.25°C (P_B_H), 5.61°C (P_A_H) and 7.63°C (NH) higher than the control (NN). The average canopy relative humidity of the primed pots was 54.83% (NN), 59.45% (NH), 60.34% (P_S_), 58.73% (P_B_), and 57.96% (P_A_) in the 2015-2016 season and 60.56% (NN), 67.32% (NH), 56.45% (P_S_H), 59.52% (P_B_H), and 61.52% (P_A_H) in the 2016-2017 season. The daily minimum and maximum temperatures, as well as the air temperature of the canopy during the thermo-stress/severe heat stress treatments, during the two growing seasons are shown in **Figure 2**. The experiment was laid out in a complete block design with a factorial arrangement. The two wheat cultivars and five temperature treatments (NN, NH, P_S_H, P_B_H, and P_A_H) were arranged as the first and second independent factors, respectively. Each treatment contained 30 replicates (pots). To avoid drought stress, we supplied sufficient water to the pots during growth and the high-temperature treatments.

### Sampling Method and Physiological Measurements

Samples and measurements were collected after heat priming at the stem-elongation, booting and anthesis stages, as well as at 5-day intervals from 15 DAA (i.e., the beginning of heat stress during grain filling) to maturity. The last fully expanded leaves were used for physiological analyses and leaf gas-exchange measurements during heat priming at the stem-elongation stage and booting. Flag leaves were used for heat priming at anthesis and heat-stress treatments during grain filling. All measurements and samplings were performed in three randomly selected pots/replicates for each treatment. We tagged uniform tillers that were flowering on the same day for sampling and measurement. Twenty stems from three pots per treatment were collected at each sampling time. The last fully expanded leaves were detached and frozen in liquid nitrogen for 1 h and then stored at -80°C. One-pot plants were used only once for measurement and sampling. All plant sampling and measurements were carried out from 9:00 to 11:00 a.m.

#### Grain Yield and Above-Ground Dry Matter Production

Spikes from three randomly selected pots were cut with scissors at soil level and threshed carefully. The grains were oven dried and weighed to measure grain yield at 14% moisture. Whole plants from another three pots were manually cut using scissors at soil level after each heat-priming treatment and at maturity. Samples were oven-dried at 105°C for 30 min and then at 70°C for 2 days to reach a constant weight for measuring the total above-ground dry matter per pot.

#### Leaf Gas-Exchange Measurement

Gas-exchange measurements using the last fully expanded leaves (five leaves per pot) were obtained on a sunny day at 09:30-11:00 a.m. using a portable photosynthesis device (LI-6400, Li-Cor Inc., United States). The chamber was equipped with a red/blue light-emitting diode (LED) light source (LI6400-02B). During the measurements, the environmental conditions were recorded according to Fan et al. (2015). The measured gas-exchange parameters were the light-saturated net CO_2_ assimilation rate (Pn), stomatal conductance (Gs), and the transpiration rate (Tr).

#### Chlorophyll Content, Soluble Protein Content, Sucrose Content and Sucrose-Phosphate Synthase Activity

Chlorophyll contents were measured according to Arnon (1949) and soluble protein contents according to Bradford (1976). The sucrose content was evaluated according to Ahmadi and Baker (2001). Sucrose-phosphate synthase (SPS, EC 2.4.1.14) activity was determined according to Pelleschi et al. (1997).

#### Superoxide Anion Radical Production Rate, Malondialdehyde Content, and Antioxidant Enzyme Activity

The rate of superoxide anion radical ($O_{2}^{•–}$) production was measured according to Sui et al. (2007). The malondialdehyde (MDA) content was assessed according to Zheng et al. (2009). SOD (EC 1.15.1.1) activity was measured according to Fisher et al. (2007), and POD (EC 1.11.1.7) activity was determined according to Zheng et al. (2009). The above measurements were carried out in three biological replicates (leaves).

### Statistical Analysis

Two-way analysis of variance (ANOVA) was performed for Pn, Gs, Tr, the chlorophyll content, the soluble protein content, the sucrose content, SPS activity, and oxidative metabolism measurements to assess significant differences between the cultivars and temperature treatments. Three-way ANOVA was performed on grain yield and its components, leaf area and above-ground dry matter to identify significant differences between the cultivars, temperature treatments and years. The statistical analyses were conducted using SPSS statistical software (SPSS ver. 10, SPSS, Chicago, IL, United States).

## Results

### Grain Yield

P_S_H treatment significantly (*P* < 0.05) decreased the spike number compared with the control (NN) for both cultivars during the 2 years (**Table 2**). Compared with NN, primed plants (P_S_H, P_B_H, and P_A_H) showed a significant decrease in kernel number for both cultivars during the 2 years; P_A_H showed the highest reduction and P_S_H the lowest reduction, and Yannong 19 exhibited a greater reduction than Yangmai 18. For both cultivars, NH significantly decreased the 1000-kernel weight compared with NN. Although the 1000-kernel weight in P_S_H and P_B_H were significantly higher than that of NH for both cultivars during the 2 years, P_B_H showed a greater increase than did P_S_H. NH significantly decreased the grain yield compared with NN for both cultivars during the 2 years, with Yannong 19 showing a greater grain yield reduction than Yangmai 18. P_S_H and P_B_H primed plants showed higher grain yields than NH, and the increases were higher in P_B_H than in P_S_H. There was significant difference in the grain yield between years, cultivars and heat treatments (*P* < 0.01). However, no significant interactions were found in the grain yield between the years, cultivars and the heat treatments. These results indicate that compared with the damage observed in non-primed plants, the grain-yield damage in P_S_H and P_B_H caused by heat stress during grain filling was alleviated via increases in the 1000-kernel weight.

**Table 2:** Effects of heat priming and post-anthesis heat stress on grain yield and its components in Yangmai 18 and Yannong 19 from 2015 to 2017.

Cultivar	Treatment	Spikes pot^-1^	Kernels spike^-1^	1000-kernel weight (g)	Yield (g pot^-1^)
**2015-2016**					
Yangmai 18	NN	23.67 bc	55.51 a	42.80 a	56.19 a
	NH	23.33 c	54.80 ab	35.16 fg	43.25 d
	P_S_H	21.67 d	53.98 bc	37.75 cd	45.67 c
	P_B_H	22.67 cd	53.26 c	38.53 c	46.08 c
	P_A_H	23.67 bc	51.65 d	36.58 de	43.74 d
Yannong 19	NN	25.33 a	51.64 d	40.68 b	53.67 b
	NH	25.00 ab	50.67 d	32.18 h	38.24 f
	P _S_H	22.67 cd	49.39 e	35.64 ef	40.85 e
	P _B_H	22.67 cd	48.26 e	36.13 ef	39.64 e
	P _A_H	25.00 ab	46.44 f	34.23 g	37.83 f
**2016-2017**					
Yangmai 18	NN	25.67 bcd	56.95 a	43.36 a	61.05 a
	NH	26.33 abc	56.14 ab	36.62 e	46.47 de
	P _S_H	23.67 e	54.94 bc	38.14 cd	48.54 cd
	P _B_H	24.67 cde	54.18 c	38.93 c	49.86 c
	P _A_H	26.00 abc	50.89 e	37.06 de	45.72 ef
Yannong 19	NN	27.00 ab	52.88 d	41.21 b	56.50 b
	NH	27.67 a	51.61 de	33.60 f	41.00 de
	P _S_H	24.00 de	49.51 f	36.10 e	43.20 fg
	P_B_H	25.67 bcd	49.32 f	36.60 e	44.74 ef
	P_A_H	27.67 a	45.40 g	34.67 f	40.12 h
*F*-value	*F*-_Y ear(Y)_	87.419^∗∗^	11.554^∗∗^	12.652^∗∗^	116.591^∗∗^
	*F*-_Cultivar(C)_	21.855^∗∗^	666.300^∗∗^	165.224^∗∗^	292.626^∗∗^
	*F*-_Treatment(T)_	18.981^∗∗^	112.532^∗∗^	195.106^∗∗^	344.941^∗∗^
	*F*-_Y × C_	0.002	0.759	0.001	0.220
	*F*-_Y × T_	0.832	4.847^∗∗^	1.106	1.982
	*F*-_C × T_	0.870	1.845	0.779	1.905
	*F*-_Y × C × T_	0.378	0.188	0.005	0.866

*NN refers to no heat priming + no heat stress at the grain-filling stage (control); NH refers to no heat priming + heat stress at the grain-filling stage; P_*S*_H refers to heat priming at the stem-elongation stage + heat stress at the grain-filling stage; P_B_H refers to heat priming at booting + heat stress at the grain-filling stage; P_*A*_H refers to heat priming at anthesis + heat stress at the grain-filling stage. F_*Y* × *C*_, F_*Y* × *T*_, F_*C* × *T*_, and F_*Y* × *C* × *T*_ refer to F-values for interactions of year with cultivar, year with treatment, cultivar with treatment, year with cultivar and treatment, respectively. ^∗∗^ indicates significant differences at the 0.01 level. The data are means ± SE (n = 3). Lowercase letters following the data within the same column refer to significant differences (P < 0.05)*.

### Above-Ground Dry Matter (DM) Production

Just after each priming (at stem elongation, booting and anthesis), DM of P_S_, P_B_, and P_A_ was significantly reduced compared to that of NN; decreases were greatest in P_A_ and lowest in P_S_ for both cultivars during the 2 years (**Table 3**). At maturity, DM values in NH, P_S_H, P_B_H, and P_A_H were significantly lower than that in NN; however, the decrease in DM was smaller in primed plants (P_S_H and P_B_H) than in non-primed plants (NH). Among the primed treatments, P_B_H showed the highest DM at maturity for both cultivars, except for Yannong 19 in 2016-2017. Compared with NN, Yannong 19 showed a greater decrease in DM than Yangmai 18. There was significant difference in the DM between cultivars and heat treatments (*P* < 0.01) at each time measurement. These results illustrate that heat priming during the stem-elongation stage and booting enhances the ability of plants to alleviate the effects of heat stress during the grain-filling stage by attenuating dry matter reduction, which would contribute to higher grain weight.

**Table 3:** Effects of heat priming and post-anthesis heat stress on above-ground dry matter production (DM; g pot^-1^) in Yangmai 18 and Yannong 19 during different growth periods from 2015 to 2017.

Cultivar	Treatment	Priming at stem-elongation	Priming at booting	Priming at anthesis	Maturity
**2015-2016**					
Yangmai 18	NN	25.29 a	65.52 a	85.60 a	131.12 a
	NH	–	–	–	96.27 e
	P _S_H	21.70 bc	60.34 c	82.30 b	105.65 c
	P _B_H	–	54.36 e	80.58 b	110.14 b
	P _A_H	–	–	67.58 e	100.86 d
Yannong 19	NN	23.37 ab	62.72 b	80.37 b	128.59 a
	NH	–	–	–	90.41 f
	P _S_H	19.49 c	56.94 d	75.51 c	92.56 f
	P _B_H	–	50.37 f	72.33 d	103.76 c
	P _A_H	–	–	59.36 f	87.63 g
**2016-2017**					
Yangmai 18	NN	27.60 a	67.59 a	90.60 a	141.12 a
	NH	–	–	–	100.27 fg
	P_S_H	23.96 b	63.22 bc	85.21 bc	111.64 d
	P_B_H	–	55.72 d	84.63 bc	116.11 c
	P_A_H	–	–	70.58 e	96.86 g
Yannong 19	NN	24.04 b	64.41 b	86.41 b	135.59 b
	NH	–	–	–	90.75 h
	P _S_H	20.07 c	61.63 c	82.75 c	105.56 e
	P _B_H	–	50.39 e	75.51 d	102.76 ef
	P _A_H	–	–	63.36 f	84.64 i
*F*-value	*F*- _Y ear(Y)_	8.672^∗^	30.398^∗∗^	114.540^∗∗^	54.567^∗∗^
	*F*-_Cultivar(C)_	34.246^∗∗^	77.348^∗∗^	241.843^∗∗^	286.698^∗∗^
	*F*-_Treatment(T)_	58.019^∗∗^	351.983^∗∗^	457.204^∗∗^	828.694^∗∗^
	*F*-_Y × C_	2.821	0.001	2.748	1.168
	*F*-_Y × T_	0.006	5.498^∗^	1.525	20.857^∗∗^
	*F*-_C × T_	0.100	2.883	6.315^∗∗^	7.657^∗∗^
	*F*-_Y × C × T_	0.003	1.477	1.702	5.343^∗∗^

*NN refers to no heat priming + no heat stress at the grain-filling stage (control); NH refers to no heat priming + heat stress at the grain-filling stage; P_*S*_H refers to heat priming at the stem-elongation stage + heat stress at the grain-filling stage; P_*B*_H refers to heat priming at booting + heat stress at the grain-filling stage; P_*A*_H refers to heat priming at anthesis + heat stress at the grain-filling stage. F_*Y* × *C*_, F_*Y* × *T*_, F_*C* × *T*_, and F_*Y* × *C* × *T*_ refer to F-values for interactions of year with cultivar, year with treatment, cultivar with treatment, year with cultivar and treatment, respectively. ^∗^ and ^∗∗^ indicate significant differences at the 0.05 and 0.01 levels, respectively. The data are means ± SE (n = 3). Lowercase letters following the data within the same column refer to significant differences (P < 0.05)*.

### Green Flag Leaf Area

There was no significant difference between treatments at the beginning of heat stress during grain filling (15 DAA; **Table 4**). However, at 20 and 25 DAA, the green flag leaf area of NH, P_S_H, P_B_H, and P_A_H was significantly decreased compared to that of NN, though the primed plants (P_S_H, P_B_H, and P_A_H) showed less of a decrease than the non-primed plants (NH). The decrease in Yannong 19 was higher than that in Yangmai 18. Among primed plants, P_S_H and P_B_H showed a higher green flag leaf area than P_A_H for both cultivars during the 2 years. There was significant difference in the green flag leaf area between cultivars and heat treatments (*P* < 0.01) at 15-25 DAA.

**Table 4:** Effects of heat priming and post-anthesis heat stress on the green flag leaf area (cm^2^ stem^-1^) of Yangmai 18 and Yannong 19 at the grain-filling stage from 2015 to 2017.

Cultivar	Treatment	15 DAA	20 DAA	25 DAA
**2015-2016**				
Yangmai 18	NN	25.60 a	21.12 a	16.65 a
	NH	25.60 a	17.27 c	8.14 d
	P _S_H	25.18 a	18.66 b	10.83 b
	P _B_H	25.66 a	19.11 b	11.48 b
	P _A_H	24.84 a	17.86 bc	9.61 c
Yannong 19	NN	20.44 b	18.59 b	11.55 b
	NH	20.44 b	14.67 d	5.65 g
	P_S_H	20.67 b	15.56 d	7.84 de
	P_B_H	20.27 b	15.76 d	6.92 ef
	P_A_H	19.72 b	14.64 d	6.12 fg
**2016-2017**				
Yangmai 18	NN	26.79 ab	21.66 a	16.95 a
	NH	26.79 ab	17.45 cd	9.03 de
	P _S_H	27.35 a	19.26 b	10.03 cd
	P _B_H	26.86 ab	18.40 bc	10.90 bc
	P _A_H	25.79 b	18.03 bc	8.63 e
Yannong 19	NN	21.39 c	19.06 b	11.76 b
	NH	21.39 c	14.70 f	5.13 g
	P_S_H	21.73 c	15.45 ef	6.10 fg
	P_B_H	20.33 d	16.42 de	6.80 f
	P_A_H	19.64 d	14.81 f	5.42 e
*F*-value	*F*-_Y ear(Y)_	38.684^∗∗^	1.103	4.989^∗^
	*F*-_Cultivar(C)_	1238.967^∗∗^	236.204^∗∗^	464.572^∗∗^
	*F*-_Treatment(T)_	8.037^∗∗^	57.793^∗∗^	205.213^∗∗^
	*F*-_Y × C_	5.924^∗^	0.056	0.881
	*F*-_Y × T_	1.752	0.217	2.646^∗^
	*F*-_C × T_	1.055	0.874	4.182^∗∗^
	*F*-_Y × C × T_	0.463	0.818	0.995

*NN refers to no heat priming + no heat stress at the grain-filling stage (control); NH refers to no heat priming + heat stress at the grain-filling stage; P_*S*_H refers to heat priming at the stem-elongation stage + heat stress at the grain-filling stage; P_*B*_H refers to heat priming at booting + heat stress at the grain-filling stage; P_*A*_H refers to heat priming at anthesis + heat stress at the grain-filling stage. F_*Y* × *C*_, F_*Y* × *T*_, F_*C* × *T*_, and F_*Y* × *C* × *T*_ refer to F-values for interactions of year with cultivar, year with treatment, cultivar with treatment, year with cultivar and treatment, respectively. ^∗^ and ^∗∗^ indicate significant differences at the 0.05 and 0.01 levels, respectively. The data are means ± SE (n = 3). Lowercase letters following the data within the same column refer to significant differences (P < 0.05)*.

### Gas Exchange

**FIGURE 3:**
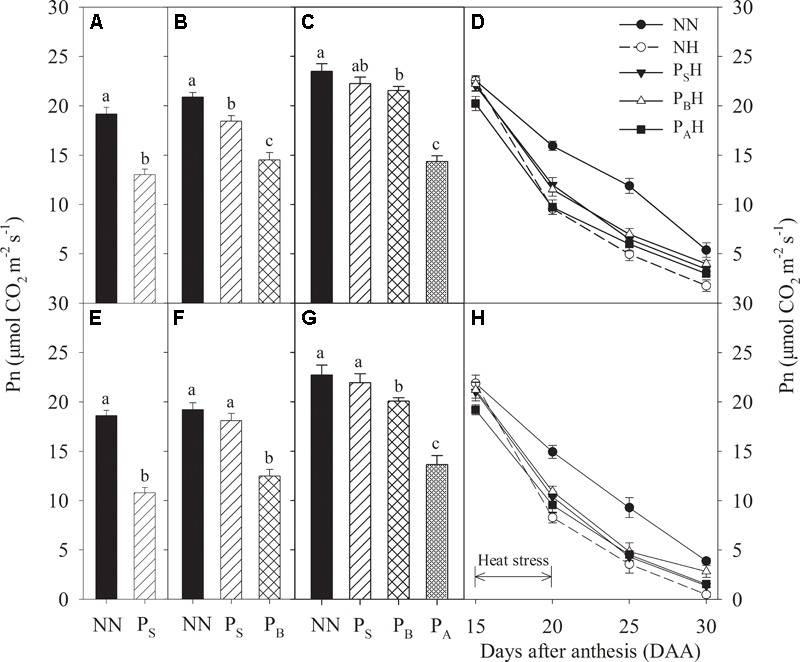
Effects of heat priming and post-anthesis heat stress on the net photosynthetic rate (Pn) in leaves of Yangmai 18 **(A–D)** and Yannong 19 **(E–H)** at the end of the heat-priming and grain-filling stages from 2016 to 2017. P_S_, P_B_, and P_A_ refer to 5 days of heat priming at the stem-elongation, booting and anthesis and stages, respectively. NN refers to no heat priming + no heat stress at the grain-filling stage (control); NH refers to no heat priming + heat stress at the grain-filling stage; P_S_H refers to heat priming at the stem-elongation stage + heat stress at the grain-filling stage; P_B_H refers to heat priming at booting + heat stress at the grain-filling stage; P_A_H refers to heat priming at anthesis + heat stress at the grain-filling stage. The data are means ± SE (*n* = 3). Lowercase letters refer to significant differences between the treatments (*P* < 0.05). Whiskers above the bars indicate the standard error.

After heat priming at the stem-elongation stage, a significant decrease in leaf Pn for both cultivars were observed for P_S_, whereas Yangmai 18 showed a smaller decrease (32.0%) than Yannong 19 (42.0%) compared with NN (**Figures 3A,E**). After heat priming at booting, P_B_ exhibited significantly decreased Pn compared with NN, and the decrease was higher in Yannong 19 (30.5%) than in Yangmai 18 (35.0%) (**Figures 3B,F**); at this time, leaf Pn in P_S_ was significantly higher than that of P_B_. After heat priming at anthesis, the leaf Pn of P_A_ was decreased by 39.0% in Yangmai 18 and 40.0% in Yannong 19 compared with NN (**Figures 3C,G**). At this stage, the leaf Pn of P_S_ and P_B_ had recovered and was significantly higher than that in P_A_. Heat stress during grain filling significantly decreased flag leaf Pn (**Figures 3D,H**). At 20 and 25 DAA, the decrease in flag leaf Pn was lower in primed plants (P_S_H, P_B_H, and P_A_H) than in non-primed plants (NH) compared with NN for both cultivars. Among the priming treatments, P_B_H showed the highest flag leaf Pn at 25 and 30 DAA.

**FIGURE 4:**
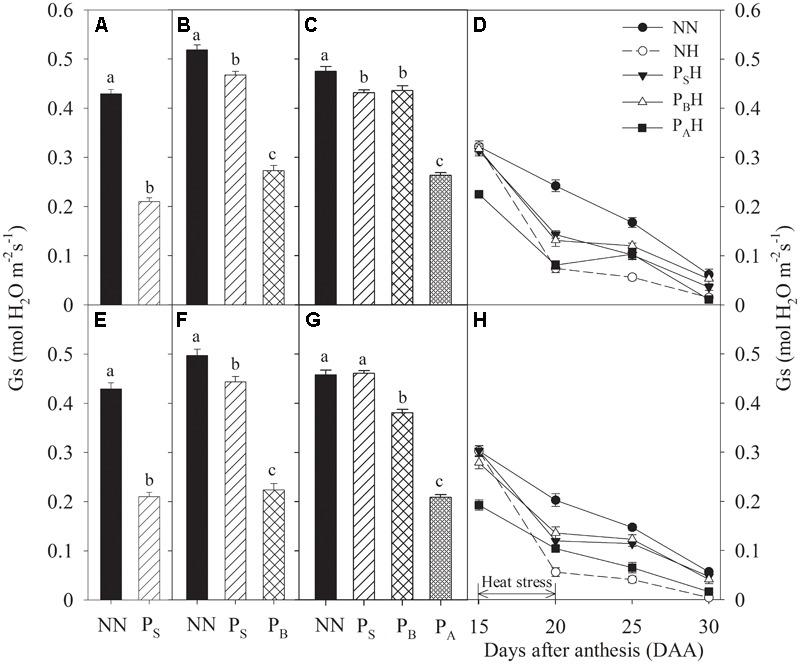
Effects of heat priming and post-anthesis heat stress on stomatal conductance (Gs) in leaves of Yangmai 18 **(A–D)** and Yannong 19 **(E–H)** at the end of the heat-priming and grain-filling stages from 2016 to 2017. P_S_, P_B_, and P_A_ refer to 5 days of heat priming at the stem-elongation, booting and anthesis stages, respectively. NN refers to no heat priming + no heat stress at the grain-filling stage (control); NH refers to no heat priming + heat stress at the grain-filling stage; P_S_H refers to heat priming at the stem-elongation stage + heat stress at the grain-filling stage; P_B_H refers to heat priming at booting + heat stress at the grain-filling stage; P_A_H refers to heat priming at anthesis + heat stress at the grain-filling stage. The data are means ± SE (*n* = 3). Lowercase letters refer to significant differences between the treatments (*P* < 0.05). Whiskers above the bars indicate the standard error.

**FIGURE 5:**
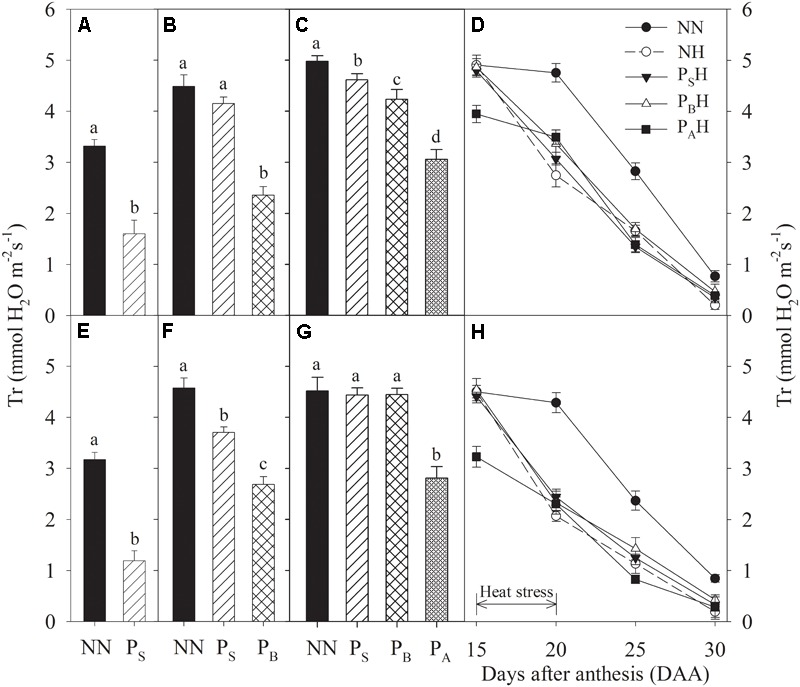
Effects of heat priming and post-anthesis heat stress on the transpiration rate (Tr) in leaves of Yangmai 18 **(A–D)** and Yannong 19 **(E–H)** at the end of the heat-priming and grain-filling stages from 2016 to 2017. P_S_, P_B_, and P_A_ refer to 5 days of heat priming at the stem-elongation, booting and anthesis stages, respectively. NN refers to no heat priming + no heat stress at the grain-filling stage (control); NH refers to no heat priming + heat stress at the grain-filling stage; P_S_H refers to heat priming at the stem-elongation stage + heat stress at the grain-filling stage; P_B_H refers to heat priming at booting + heat stress at the grain-filling stage; P_A_H refers to heat priming at anthesis + heat stress at the grain-filling stage. The data are means ± SE (*n* = 3). Lowercase letters refer to significant differences between the treatments (*P* < 0.05). Whiskers above the bars indicate the standard error.

Just after each priming, leaf Gs and Tr of P_S_, P_B_, and P_A_ were significantly reduced compared to NN, with the greatest decreases in P_A_ and the lowest in P_S_ for both cultivars during the 2 years (**Figures 4A–C,E–G, 5A–C,E–G**). At 15 DAA, P_A_H showed a significant decrease in flag leaf Gs and Tr in both cultivars compared with NN (**Figures 4D,H, 5D,H**). After the post-anthesis heat-stress treatment, the flag leaf Gs of P_S_H and P_B_H primed plants was significantly higher than that of non-primed NH plants from 20 to 30 DAA (**Figures 4D,H**), whereas the flag leaf Tr did not differ significantly between primed and non-primed plants (**Figures 5D,H**). At each time measurement, there was significant difference in the leaf Pn, Gs, and Tr between heat treatments (*P* < 0.01; **Table 5**). During stem elongation and booting, primed plants showed higher grain yield than non-primed plants, which might be related to the former retaining greater photosynthetic capacity during the stress period.

**Table 5:** Two-way ANOVA analysis for photosynthetic parameters, sucrose content and sucrose-phosphate synthase (SPS) activity of the two cultivars, as affected by heat priming and post-anthesis heat stress, and the interactive effect.

Time	Pn			Gs			Tr			Sucrose			SPS		
	C	T	C × T	C	T	C × T	C	T	C × T	C	T	C × T	C	T	C × T
Priming at stem-elongation	^∗∗^	^∗∗^	ns	ns	^∗∗^	ns	ns	^∗∗^	ns	ns	^∗∗^	ns	ns	^∗∗^	ns
Priming at booting	^∗∗^	^∗∗^	ns	^∗∗^	^∗∗^	^∗^	ns	^∗∗^	^∗^	^∗∗^	^∗∗^	ns	^∗∗^	^∗∗^	^∗∗^
Priming at anthesis	^∗∗^	^∗∗^	ns	^∗∗^	^∗∗^	^∗∗^	^∗^	^∗∗^	^∗^	^∗∗^	^∗∗^	ns	^∗∗^	^∗∗^	ns
15 DAA	^∗∗^	^∗∗^	ns	^∗∗^	^∗∗^	ns	^∗∗^	^∗∗^	ns	^∗∗^	^∗∗^	^∗∗^	^∗∗^	^∗∗^	ns
20 DAA	^∗∗^	^∗∗^	ns	^∗∗^	^∗∗^	^∗∗^	^∗∗^	^∗∗^	^∗^	ns	^∗∗^	^∗^	^∗∗^	^∗∗^	ns
25 DAA	^∗∗^	^∗∗^	ns	^∗∗^	^∗∗^	^∗∗^	^∗∗^	^∗∗^	ns	^∗∗^	^∗∗^	^∗∗^	^∗∗^	^∗∗^	ns
30 DAA	^∗∗^	^∗∗^	ns	ns	^∗∗^	^∗^	ns	^∗∗^	ns	^∗∗^	^∗∗^	^∗^	^∗∗^	^∗∗^	ns

*Pn, Gs, and Tr refer to the net photosynthetic rate, stomatal conductance and transpiration rate, respectively. DAA refers to days after anthesis. ^∗^, ^∗∗^, and ns indicate significance at 0.05 and 0.01 levels and no significant difference, respectively. C, T, and C × T refer to cultivar, treatment and the interaction of cultivar with treatment, respectively.*

### Flag Leaf Chlorophyll and Soluble Protein Contents

**FIGURE 6:**
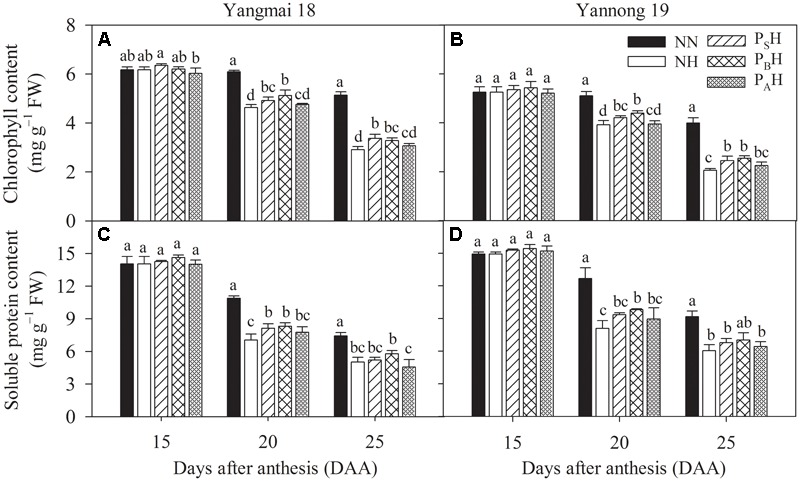
Effects of heat priming and post-anthesis heat stress on the chlorophyll **(A,B)** and soluble protein content **(C,D)** in flag leaves of Yangmai 18 and Yannong 19 at the grain-filling stage from 2016 to 2017. NN refers to no heat priming + no heat stress at the grain-filling stage (control); NH refers to no heat priming + heat stress at the grain-filling stage; P_S_H refers to heat priming at the stem-elongation stage + heat stress at the grain-filling stage; P_B_H refers to heat priming at booting + heat stress at the grain-filling stage; P_A_H refers to heat priming at anthesis + heat stress at the grain-filling stage. The data are means ± SE (*n* = 3). Lowercase letters refer to significant differences between the treatments (*P* < 0.05). Whiskers above the bars indicate the standard error.

Flag leaf chlorophyll contents between primed and non-primed plants did not differ significantly at 15 DAA (**Figures 6A,B**). At 20 and 25 DAA, chlorophyll contents in the flag leaves of NH, P_S_H, P_B_H, and P_A_H were significantly reduced compared to NN; primed plants (P_S_H and P_B_H) showed significantly higher values than non-primed (NH) plants of both cultivars, especially in the P_B_H treatment.

Post-anthesis heat stress significantly decreased the flag leaf soluble protein content, and Yannong 19 showed a greater decrease than did Yangmai 18 (**Figures 6C,D**). At 20 DAA, flag leaf soluble protein contents in P_B_H were significantly higher than those in NH for both cultivars. At 25 DAA, the flag leaf soluble protein content did not differ significantly between primed and non-primed plants of both cultivars. There was significant difference in the flag leaf chlorophyll and soluble protein content between cultivars at 15-25 DAA (*P* < 0.01; **Table 6**).

**Table 6:** Two-way ANOVA analysis for chlorophyll content, soluble protein content, superoxide anion radical ($O_{2}^{•–}$) production rate, malondialdehyde (MDA) content and antioxidant enzyme activity of the two cultivars, as affected by heat priming and post-anthesis heat stress, and the interactive effect.

Time	Chlorophyll			Soluble protein			$O_{2}^{•–}$			MDA			SOD			POD		
	C	T	C × T	C	T	C × T	C	T	C × T	C	T	C × T	C	T	C × T	C	T	C × T
15 DAA	^∗∗^	ns	ns	^∗∗^	ns	ns	ns	^∗∗^	ns	^∗∗^	^∗∗^	ns	^∗∗^	^∗∗^	ns	ns	ns	^∗^
20 DAA	^∗∗^	^∗∗^	ns	^∗∗^	^∗∗^	ns	ns	^∗∗^	ns	^∗∗^	^∗∗^	^∗^	^∗∗^	^∗∗^	ns	^∗∗^	^∗∗^	ns
25 DAA	^∗∗^	^∗∗^	ns	^∗∗^	^∗∗^	ns	^∗∗^	^∗∗^	^∗∗^	^∗∗^	^∗∗^	^∗^	^∗∗^	^∗∗^	ns	^∗∗^	^∗∗^	ns

*SOD and POD refer to superoxide dismutase activity and peroxidase activity, respectively. DAA refers to days after anthesis. ^∗^, ^∗∗^, and ns indicate significance at 0.05 and 0.01 levels and no significant difference, respectively. C, T, and C × T refer to cultivar, treatment and the interaction of cultivar with treatment, respectively*.

### Sucrose Content and SPS Activity

**FIGURE 7:**
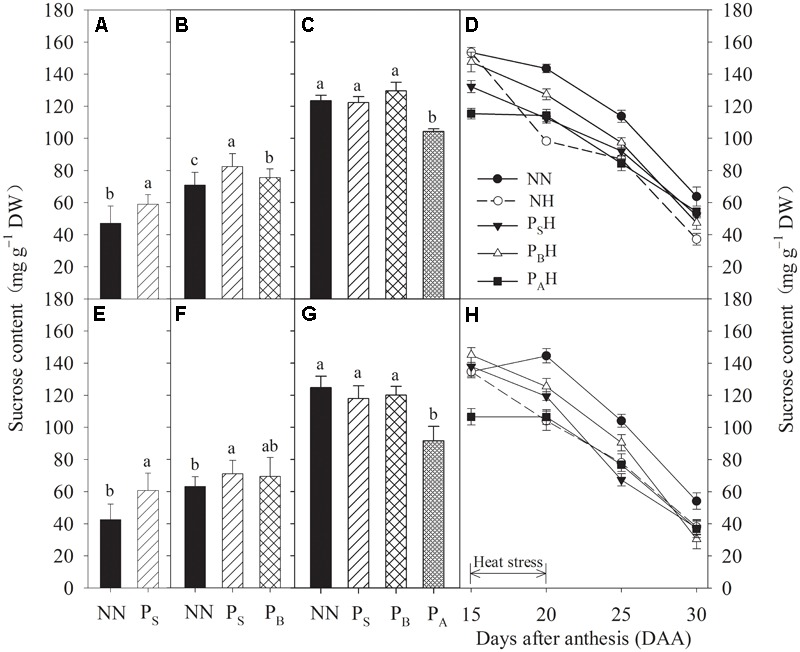
Effects of heat priming and post-anthesis heat stress on the sucrose content in leaves of Yangmai 18 **(A–D)** and Yannong 19 **(E–H)** at the end of the heat-priming and grain-filling stages from 2016 to 2017. P_S_, P_B_, and P_A_ refer to 5 days of heat priming at the stem-elongation, booting and anthesis stages, respectively. NN refers to no heat priming + no heat stress at the grain-filling stage (control); NH refers to no heat priming + heat stress at the grain-filling stage; P_S_H refers to heat priming at the stem-elongation stage + heat stress at the grain-filling stage; P_B_H refers to heat priming at booting + heat stress at the grain-filling stage; P_A_H refers to heat priming at anthesis + heat stress at the grain-filling stage. The data are means ± SE (*n* = 3). Lowercase letters refer to significant differences between the treatments (*P* < 0.05). Whiskers above the bars indicate the standard error.

After heat priming at the stem-elongation and booting stages, the sucrose content in P_S_ was significantly increased compared to that in NN for both cultivars (**Figures 7A,B,E,F**). After heat priming at anthesis, the P_A_ treatment resulted in significantly decreased sucrose contents in both cultivars, though the sucrose content did not differ significantly among P_S_, P_B_, and NN (**Figures 7C,G**). Post-anthesis heat stress decreased the flag leaf sucrose content in NH, P_S_H, P_B_H, and P_A_H from 15 to 30 DAA, but the decrease in P_B_H primed plants was significantly reduced compared to NH non-primed plants of both cultivars (**Figures 7D,H**).

**FIGURE 8:**
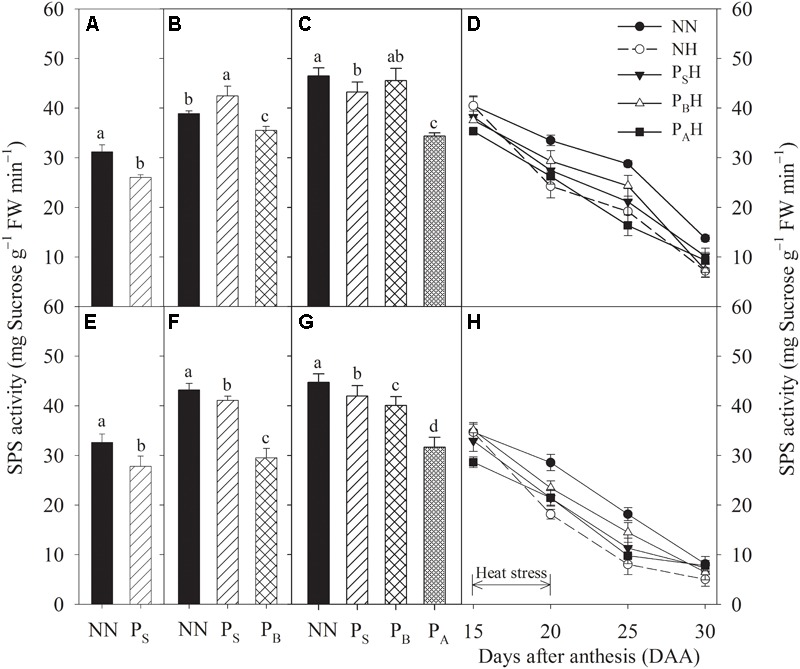
Effects of heat priming and post-anthesis heat stress on sucrose-phosphate synthase (SPS) activity in leaves of Yangmai 18 **(A–D)** and Yannong 19 **(E–H)** at the end of the heat-priming and grain-filling stages from 2016 to 2017. P_S_, P_B_, and P_A_ refer to 5 days of heat priming at the stem-elongation, booting and anthesis stages, respectively. NN refers to no heat priming + no heat stress at the grain-filling stage (control); NH refers to no heat priming + heat stress at the grain-filling stage; P_S_H refers to heat priming at the stem-elongation stage + heat stress at the grain-filling stage; P_B_H refers to heat priming at booting + heat stress at the grain-filling stage; P_A_H refers to heat priming at anthesis + heat stress at the grain-filling stage. The data are means ± SE (*n* = 3). Lowercase letters refer to significant differences between the treatments (*P* < 0.05). Whiskers above the bars indicate the standard error.

After heat priming at three growth stages, P_S_, P_B_, and P_A_ plants showed significantly decreased leaf SPS activity, and the decreases were greater in Yannong 19 than in Yangmai 18 (**Figures 8A–C,E–G**). At 15 DAA, the lowest flag leaf SPS activity for both cultivars was observed for P_A_H plants (**Figures 8D,H**). Compared with NN, heat stress during grain filling significantly decreased flag leaf SPS activity in both primed (P_S_H, P_B_H, and P_A_H) and non-primed (NH) plants from 20 to 25 DAA. At 20 and 25 DAA, P_B_H showed the highest flag leaf SPS activity among the primed treatments for both cultivars compared with NH. At each time measurement, there was significant difference in the leaf sucrose content and SPS activity between heat treatments (*P* < 0.01; **Table 5**). These results indicate that compared to NH, P_B_H treatment maybe enhanced the ability to regulate the transformation of photosynthetic products into sucrose in wheat leaves during grain filling after heat stress, which resulted in a higher carbohydrate supply for the grain.

### $O_{2}^{•–}$ Production Rate, MDA Content, and Antioxidant Enzyme Activity

**FIGURE 9:**
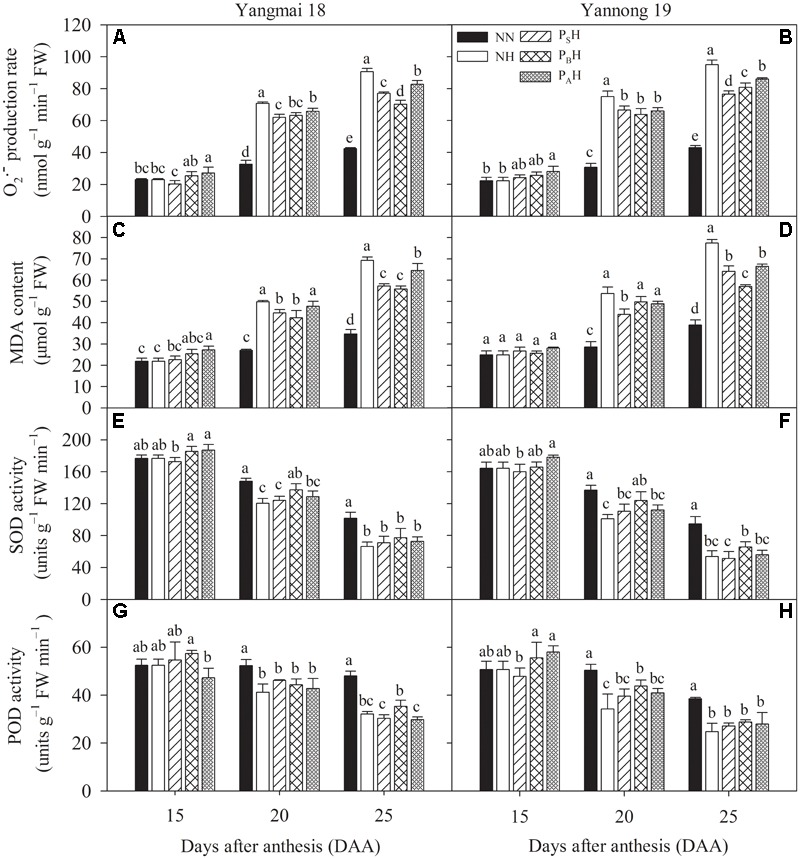
Effects of heat priming and post-anthesis heat stress on the superoxide anion radical ($O_{2}^{•–}$) production rate **(A,B)**, malondialdehyde (MDA) content **(C,D)**, superoxide dismutase (SOD) activity **(E,F)**, peroxidase (POD) activity **(G,H)** in flag leaves of Yangmai 18 and Yannong 19 at the grain-filling stage from 2016 to 2017. NN refers to no heat priming + no heat stress at the grain-filling stage (control); NH refers to no heat priming + heat stress at the grain-filling stage; P_S_H refers to heat priming at the stem-elongation stage + heat stress at the grain-filling stage; P_B_H refers to heat priming at booting + heat stress at the grain-filling stage; P_A_H refers to heat priming at anthesis + heat stress at the grain-filling stage. The data are means ± SE (*n* = 3). Lowercase letters refer to significant differences between the treatments (*P* < 0.05). Whiskers above the bars indicate the standard error.

At 15 DAA, P_A_H showed a significantly increased flag leaf $O_{2}^{•–}$ production rate for both cultivars compared with NN (**Figures 9A,B**). Heat stress during grain filling significantly increased flag leaf $O_{2}^{•–}$ production in primed plants (P_S_H, P_B_H, and P_A_H) as well as non-primed plants (NH) at 20 and 25 DAA compared with NN, with higher increases for Yannong 19 than Yangmai 18 and in non-primed than in primed plants. At 25 DAA, the flag leaf $O_{2}^{•–}$ production rate in P_S_H and P_B_H plants was significantly lower than that in P_A_H plants. The same trend was observed for the MDA content (**Figures 9C,D**). This indicated that post-anthesis heat stress significantly damaged the flag leaf cell membrane, as P_S_H and P_B_H primed plants showed reduced levels of membrane lipid oxidation in flag leaves compared with NH. There was significant difference in the flag leaf $O_{2}^{•–}$ production rate and MDA content between heat treatments at 15-25 DAA (*P* < 0.01; **Table 6**).

At 20 and 25 DAA, heat stress during grain filling decreased flag leaf SOD activity in primed and non-primed plants compared with NN plants (**Figures 9E,F**). Flag leaf SOD activity was higher in P_B_H primed plants than in NH non-primed plants for both cultivars, and the difference was significant at 20 DAA. Heat stress during the grain-filling period also significantly decreased flag leaf POD activity in primed (P_S_H, P_B_H, and P_A_H) and non-primed (NH) plants at 20 and 25 DAA compared with NN plants, and the decrease was higher in non-primed than in primed plants (**Figures 9G,H**). P_B_H treatment significantly increased flag leaf POD activity in Yannong 19 at 20 DAA compared with NH. The higher antioxidant capacity and lower levels of $O_{2}^{•–}$ production and MDA content identified in primed plants (especially P_B_H) than in non-primed plants indicated their enhanced redox defense response and capacity to scavenge ROS by down-regulating the peroxidation of cell membrane lipids during heat stress.

## Discussion

The present study showed that heat-primed wheat plants displayed a positive response to subsequent high temperature stress that effectively maintained growth and alleviated the damage associated with post-anthesis heat stress. Indeed, the results demonstrated that heat-priming treatments P_S_H and P_B_H tempered the reduction in grain yield caused by post-anthesis heat stress compared with non-primed (NH) plants, primarily due to increased 1000-kernel weight (**Table 2**). The optimal temperature of vernal-type winter wheat is higher than that of facultative type wheat during the vernalization phase; the vernal type is better adapted to warm conditions (Fan et al., 2015) and thus shows higher tolerance to heat stress. Heat-priming treatments in the present study more clearly reduced the yield loss caused by post-anthesis heat stress in vernal-type Yangmai 18 than in facultative type Yannong 19. The results for the two genotypes were similar to those of our previous study (Fan et al., 2015, 2017). The present study showed that after priming at the stem-elongation and booting stages, above-ground DM production by the two wheat cultivars decreased, though this was largely recovered as growth continued (**Table 3**). Grain is mostly formed from carbohydrates stored before anthesis and photosynthetic production during grain filling (Tian et al., 2012). Our results showed that post-anthesis heat stress significantly decreased wheat DM at maturity, which was lower in P_S_H and P_B_H primed plants than in non-primed NH plants during both study years (**Table 3**). Wang et al. (2012) found that under post-anthesis heat stress, pre-anthesis high-temperature acclimation promotes remobilization of reserved carbohydrates from the stems to the grains in winter wheat compared to non-acclimated plants by enhancing the activities of fructan-catalytic enzymes. This effect likely underlies the increase in post-anthesis above-ground DM in the primed treatments compared with that in the non-primed treatment, contributing to higher grain weight. Therefore, heat priming during the stem-elongation and booting stages proved to contribute greatly to grain yield sustainability by enhancing the ability of plants to mitigate the effects of heat stress during the grain-filling stage.

Sucrose is the main product of plant photosynthesis, and imbalance occurs between photoassimilate accumulation and utilization due to photorespiration, leading to a reduction in photoassimilate translocation to reproductive organs (Zahoor et al., 2017). The present study showed higher sucrose contents compared to NN plants in P_S_ and P_B_ plants after heat priming at the stem-elongation and booting stages (**Figures 7A,B,E,F**). High nighttime temperature increases plant respiration and carbohydrate utilization efficiency for plant growth. Chen et al. (2014) reported that stimulated nighttime depletion of leaf carbohydrates under night warming is compensated for by enhanced daytime production. After heat priming, the increased sucrose content in P_S_ and P_B_ plants may have been due to enhanced carbohydrate accumulation and growth activity during heat priming. Sucrose accumulation in the flag leaf after anthesis, which contributes to grain filling, tends to be reduced by heat stress, but P_S_H and P_B_H plants showed a higher sucrose content than non-primed NH plants (**Figures 7D,H**), possibly maintaining a higher carbohydrate supply for the grain (Talukder et al., 2013). The activity of SPS plays a key role in regulating the transformation of photosynthetic products into sucrose in wheat leaves. High temperatures during the middle and late stages of grain filling can reduce the activity of the key enzyme SPS, which potentially limits photosynthesis in wheat (Zhao et al., 2008). The present study showed that compared with non-primed NH plants, primed P_S_H and P_B_H plants had higher flag leaf SPS activity after heat stress, which may regulate the transformation of photosynthetic products into sucrose and contribute to increased photosynthesis. In addition, the positive influence of heat priming on tolerance to heat stress during grain filling was more significant in plants primed at the booting stage than in those primed at the stem-elongation stage or later at anthesis.

Many studies have demonstrated that most plant species can adapt to alterations in growth temperature by adjusting the photosynthetic system to optimize performance in a new temperature environment (Sharkey, 2005; Atkin et al., 2006; Kurek et al., 2007; Martinez et al., 2014). The present study showed that after heat priming, P_S_, P_B_, and P_A_ plants all exhibited decreased leaf Pn compared with NN; however, leaf Pn recovered in P_S_ and P_B_ plants after normal temperatures were restored (**Figures 3B,C,F,G**). This suggests that increasing the temperature by 5°C during early reproductive stages causes no irreversible damage to PSII of winter wheat. After post-anthesis heat stress, the primed plants showed a superior ability compared with non-primed plants to develop grains with a higher weight; this occurred through a sustained a higher rate of carbon accumulation through Pn during the grain-filling period (**Figures 3D,H**). Havaux (1993) indicated that exposing potato leaves to 35°C for 20 min significantly enhanced the stability of PSII to subsequent heat stress (>42°C). Maize plants grown at elevated temperatures also exhibit greater resistance to heat stress and better post-stress recovery than control plants grown at ambient temperature (Sinsawat et al., 2004). This effect might be due to adaptation of the plants to the higher temperature via the improved heat stability of PSII. The photosynthetic apparatus of vernal-type Yangmai 18 showed a slightly higher tolerance to heat stress and a better recovery than facultative type Yannong 19 (**Figure 3**). Notably, higher Gs and Tr in flag leaves after post-anthesis heat stress were observed in primed plants (especially P_S_H and P_B_H) compared to non-primed plants NH (**Figures 4, 5**). This finding might suggest that the P_S_H and P_B_H primed plants better protected leaf function by modulating stomatal opening or by regulating non-stomatal processes during the later, more-severe high-temperature stress (Li et al., 2014a). During early reproductive stages, the primed plants showed higher grain yield than the non-primed plants, which might be related to the capability of the former to retain more photosynthetic substrates (chlorophyll) during the period of stress (Abid et al., 2016). The leaf chlorophyll content during the grain-filling stage has been used as an efficient indicator of photosynthetic ability and stress tolerance in plants (Wang et al., 2011). After heat stress during grain filling, the primed plants (especially P_B_H) showed higher chlorophyll contents than did the non-primed NH plants (**Figures 6A,B**), which limited the reduction in photochemical activity of chloroplasts. This finding indicates that compared with non-primed treatment, heat priming at the booting stage enhanced photosynthetic capacity and stress tolerance in the flag leaves of winter wheat, which benefited the grain-filling process.

Post-anthesis heat stress significantly reduced the green flag leaf area of both wheat cultivars, though the P_S_H and P_B_H primed plants showed a significantly greater green flag leaf area than the non-primed NH plants (**Table 4**). A larger leaf area results in a slower senescence rate, which can enhance canopy light interception and photosynthetic efficiency and may contribute to increased dry matter accumulation and grain filling (Chen et al., 2014). This appears to be the reason for the increase in post-anthesis photosynthetic products and source activity improvement caused by heat priming during the stem-elongation stage and booting and may have also contributed to the higher grain weight. The soluble protein content in plant leaves is reflected in the nitrogen content, which has been positively correlated with photosynthetic capacity (Zhao et al., 2008). In the present study, the flag leaf soluble protein content in the P_B_H primed plants was significantly higher than in the non-primed NH plants after post-anthesis heat stress (**Figures 6C,D**). This indicated that heat priming at the booting stage was able to mitigate the decrease in soluble protein content under post-anthesis heat stress, increasing the photosynthetic capacity and grain yield. The above evidence suggests that compared to non-priming, heat priming during stem elongation and booting can alleviate grain yield damage caused by post-anthesis heat stress by promoting carbohydrate storage before anthesis and an increase in photosynthetic products during the grain-filling stage of winter wheat.

Pre- and post-anthesis heat stress has already been shown to reduce photosynthetic rates in wheat through oxidative damage, which accelerates leaf senescence (Wang et al., 2011). Under heat-stress conditions, higher photosystem efficiency also prevents ROS generation and assists the rapid and complete photosystem recovery at normal temperatures (Sharkey, 2005; Li et al., 2014a). In the present study, we found that P_S_H and P_B_H primed plants had a lower flag leaf $O_{2}^{•–}$ production rate than did non-primed NH plants and that the decrease was higher in Yannong 19 than in Yangmai 18 (**Figures 9A,B**). In addition, leaf SOD activity was higher in primed plants (especially P_B_H) than in non-primed NH plants for both cultivars (**Figures 9E,F**), consistent with the $O_{2}^{•–}$ production rates. This result indicates that heat priming during the stem-elongation stage and booting maintained a sufficient antioxidant capacity to avoid ROS accumulation, thereby delaying leaf senescence. Ristic et al. (2007) indicated that maintenance of high antioxidant capacity to alleviate cell membrane damage and lipid peroxidation is closely related to heat tolerance in plants. MDA has been used as an indicator of free radical damage to cell membranes and membrane thermo-stability caused by heat stress (Wahid et al., 2007), and the lower MDA content observed after post-anthesis heat stress in P_B_H plants demonstrates the enhanced ROS-scavenging capacity of the antioxidant system (**Figures 9C,D**). This result indicated that P_B_H plants maintained higher cell membrane thermo-stability (an essential trait related to heat stress tolerance) compared with non-primed plants undergoing heat stress during grain filling. This finding suggests that in addition to promoting PSII activity, heat priming during booting enhanced antioxidant capacity and reduced the risk of membrane injury in winter wheat under a later severe high-temperature stress during grain filling. The higher SOD activity and lower MDA content identified in primed plants showed their enhanced redox defense and ability to scavenge ROS by down-regulating peroxidation of cell membrane lipids during heat stress.

Moreover, our experiments were conducted to test the hypothesis that plants pre-exposed to heat stress can better tolerate subsequent severe heat stress compared to plants without pre-heat exposure. Controlled conditions were provided to plants to minimize the environmental effect and precisely apply the treatments. As it was difficult to avoid exposing roots to the heat stress, both shoots and roots (inside the pots) were subjected to this stress; nonetheless, similar conditions were provided to both the primed and non-primed wheat plants. Obviously, this would not be the case for plant production in the field.

## Conclusion

**FIGURE 10:**
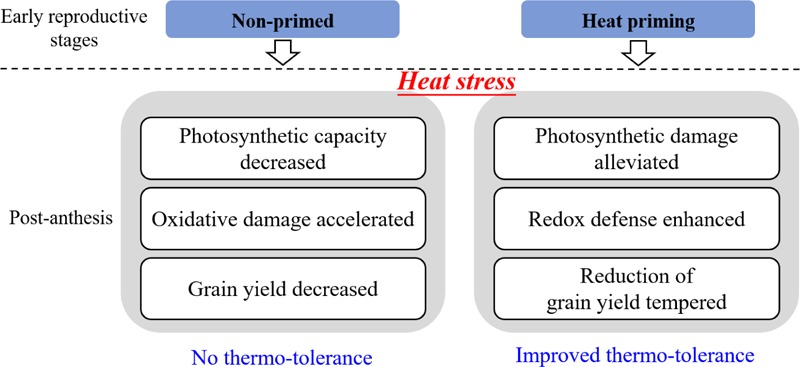
A model in comparison the behavior of the plants under heat priming with the non-primed ones.

Wheat plants pre-exposed to moderate high-temperature stress retained a heat-stress memory that triggered stress-scavenging mechanisms during severe high-temperature stress post-anthesis. Plants subjected to heat priming during early reproductive stages such as stem elongation and booting showed enhanced photosynthetic capacity via increases in green flag leaf area, elevated chlorophyll and soluble protein contents, greater photoprotection and more efficient antioxidant enzyme systems, thereby improving source productivity and leading to a greater carbohydrate supply and less grain yield reduction than in non-primed plants. Heat priming during early reproductive growth stages (especially at booting) proved to be a valuable strategy for triggering plants to initialize an efficient tolerance mechanism, which in turn permitted the plants to tolerate subsequent, more-severe high-temperature conditions (**Figure 10**). Thus, knowledge of the mechanisms underlying temperature adaptation and acclimation in wheat cultivars offers novel perspectives for understanding how crop performance can be improved under changing climate conditions.

## Author Contributions

YF, CM, ZH, and TD designed the experiment. YF conducted the study, collected and analyzed the data, and prepared the manuscript draft. DJ and XH helped in sampling and physiological parameter measurements. WZ and SM helped in drafting the manuscript and the interpretation of the results. SJ and MA assisted in manuscript writing and editing.

## Conflict of Interest Statement

The authors declare that the research was conducted in the absence of any commercial or financial relationships that could be construed as a potential conflict of interest.

## References

[B1] Abid M. Tian Z. Ata-Ul-Karim S. T. Yang L. Cui Y. Zahoor R. (2016). Improved tolerance to post-anthesis drought stress by pre-drought priming at vegetative stages in drought-tolerant and -sensitive wheat cultivars. *Plant Physiol. Biochem.* 106 218–227. 10.1016/j.plaphy.2016.05.003 27179928

[B2] Ahmadi A. Baker D. A. (2001). The effect of water stress on the activities of key regulatory enzymes of the sucrose to starch pathway in wheat. *Plant Growth Regul.* 35 81–91. 10.1023/A:1013827600528

[B3] Arnon D. I. (1949). Copper enzymes in isolated chloroplast. Polyphenoloxidase in *Beta vulgaris*. *Plant Physiol.* 24 1–15. 10.1104/pp.24.1.1 16654194 PMC437905

[B4] Asseng S. Foster I. Turner N. C. (2011). The impact of temperature variability on wheat yields. *Glob. Change Biol.* 17 997–1012. 10.1111/j.1365-2486.2010.02262.x

[B5] Atkin O. K. Scheurwater I. Pons T. L. (2006). High thermal acclimation potential of both photosynthesis and respiration in two lowland *Plantago* species in contrast to an alpine congeneric. *Glob. Change Biol.* 12 500–515. 10.1111/j.1365-2486.2006.01114.x

[B6] Backhaus S. Kreyling J. Grant K. Beierkuhnlein C. Walter J. Jentsch A. (2014). Recurrent mild drought events increase resistance toward extreme drought stress. *Ecosystems* 17 1068–1081. 10.1007/s10021-014-9781-5

[B7] Bradford M. M. (1976). A rapid and sensitive method for the quantitation of microgram quantities of protein utilizing the principle of protein-dye binding. *Anal. Biochem.* 72 248–254. 10.1016/0003-2697(76)90527-3 942051

[B8] Chen J. Tian Y. Zhang X. Zheng C. Song Z. Deng A. (2014). Nighttime warming will increase winter wheat yield through improving plant development and grain growth in north China. *J. Plant Growth Regul.* 33 397–407. 10.1007/s00344-013-9390-0

[B9] Dias A. S. Semedo J. Ramalho J. C. Lidon F. C. (2011). Bread and durum wheat under heat stress: a comparative study on the photosynthetic performance. *J. Agron. Crop Sci.* 197 50–56. 10.1111/j.1439-037X.2010.00442.x

[B10] Fan Y. Tian M. Jing Q. Tian Z. Han H. Jiang D. (2015). Winter night warming improves pre-anthesis crop growth and post-anthesis photosynthesis involved in grain yield of winter wheat (*Triticum aestivum* L.). *Field Crop. Res.* 178 100–108. 10.1016/j.fcr.2015.04.001

[B11] Fan Y. Tian Z. Yan Y. Hu C. Abid M. Jiang D. (2017). Winter night-warming improves post-anthesis physiological activities and sink strength in relation to grain filling in winter wheat (*Triticum aestivum* L.). *Front. Plant Sci.* 8:992. 10.3389/fpls.2017.00992 28659943 PMC5469006

[B12] Farooq M. Kobayashi N. Ito O. Wahid A. Serraj R. (2010). Broader leaves result in better performance of indica rice under drought stress. *J. Plant Physiol.* 167 1066–1075. 10.1016/j.jplph.2010.03.003 20392520

[B13] Fisher R. A. Tanabe S. Buxton D. B. Olson M. S. (2007). Ultraviolet-B irradiation-induced freezing tolerance in relation to antioxidant system in winter wheat (*Triticum aestivum* L.) leaves. *Environ. Exp. Bot.* 60 300–307. 10.1016/j.envexpbot.2006.12.003

[B14] Food and Agriculture Organization [FAO] (2013). *FAOSTAT.* Rome: FAO.

[B15] Havaux M. (1993). Characterization of thermal damage to the photosynthetic electron transport system in potato leaves. *Plant Sci.* 94 19–33. 10.1016/0168-9452(93)90003-I

[B16] Intergovernmental Panel on Climate Change [IPCC] (2014). *Climate Change 2013. The Physical Science Basis: Working Group I Contribution to the Fifth Assessment Reportsta of the Intergovernmental Panel on Climate Change.* Cambridge: Cambridge University Press.

[B17] Jenner C. F. (1994). Starch synthesis in the kernel of wheat under high temperature conditions. *Funct. Plant Biol.* 21 791–806. 10.1007/s11627-018-9893-2 29780215 PMC5954006

[B18] Kurek I. Chang T. K. Bertain S. M. Madrigal A. Liu L. Lassner M. W. (2007). Enhanced thermostability of *Arabidopsis* Rubisco activase improves photosynthesis and growth rates under moderate heat stress. *Plant Cell* 19 3230–3241. 10.1105/tpc.107.054171 17933901 PMC2174701

[B19] Li J. Qiu Z. Zhang X. Wang L. (2011). Exogenous hydrogen peroxide can enhance tolerance of wheat seedlings to salt stress. *Acta Physiol. Plant.* 33 835–842. 10.1016/j.ecoenv.2014.03.014 24726929

[B20] Li X. Cai J. Liu F. Dai T. Cao W. Dong J. (2014a). Cold priming drives the sub-cellular antioxidant systems to protect photosynthetic electron transport against subsequent low temperature stress in winter wheat. *Plant Physiol. Biochem.* 82 34–43. 10.1016/j.plaphy.2014.05.005 24887010

[B21] Li X. Cai J. Liu F. Dai T. Cao W. Jiang D. (2014b). Physiological, proteomic and transcriptional responses of wheat to combination of drought or waterlogging with late spring low temperature. *Funct. Plant Biol.* 41 690–703. 10.1071/FP13306 32481024

[B22] Lobell D. B. Schlenker W. Costa-Roberts J. (2011). Climate trends and global crop production since 1980. *Science* 333 616–620. 10.1126/science.1204531 21551030

[B23] Lobell D. B. Sibley A. Ortiz-Monasterio J. I. (2012). Extreme heat effects on wheat senescence in India. *Nat. Clim. Chang.* 2 186–189. 10.1038/nclimate1356

[B24] Martinez C. A. Bianconi M. Silva L. Approbato A. Lemos M. Santos L. (2014). Moderate warming increases PSII performance, antioxidant scavenging systems and biomass production in *Stylosanthes capitata* Vogel. *Environ. Exp. Bot.* 102 58–67. 10.1016/j.envexpbot.2014.02.001

[B25] Masoni A. Ercoli L. Mariotti M. Arduini I. (2007). Post-anthesis accumulation and remobilization of dry matter, nitrogen and phosphorus in durum wheat as affected by soil type. *Eur. J. Agron.* 26 179–186. 10.1016/j.eja.2006.09.006

[B26] Mohammed A. R. Tarpley L. (2010). Effects of high night temperature and spikelet position on yield-related parameters of rice (*Oryza sativa* L.) plants. *Eur. J. Agron.* 33 117–123. 10.1016/j.eja.2009.11.006

[B27] Moriondo M. Giannakopoulos C. Bindi M. (2011). Climate change impact assessment: the role of climate extremes in crop yield simulation. *Clim. Change* 104 679–701. 10.1016/j.scitotenv.2018.02.322 29554771

[B28] Mu C. Yang X. Yang J. Li K. Zheng D. (2015). Freezing resistance and injury indices for different cultivars of winter-spring wheat in Huang-Huai-Hai Plain. I Comparison of freezing resistance for different cultivars of winter-spring wheat during mid-winter period. *Chin. J. Appl. Ecol.* 26 3119–3125. 26995921

[B29] Pelleschi S. Rocher J. P. Prioul J. L. (1997). Effect of water restriction on carbohydrate metabolism and photosynthesis in mature maize leaves. *Plant Cell Environ.* 20 493–503. 10.1046/j.1365-3040.1997.d01-89.x

[B30] Porter J. R. Gawith M. (2015). Temperatures and the growth and development of wheat: a review. *Eur. J. Agron.* 10 23–36. 10.1016/S1161-0301(98)00047-1

[B31] Ristic Z. Bukovnik U. Prasad P. V. (2007). Correlation between heat stability of thylakoid membranes and loss of chlorophyll in winter wheat under heat stress. *Crop Sci.* 47 2067–2073. 10.2135/cropsci2006.10.0674

[B32] Sharkey T. D. (2005). Effects of moderate heat stress on photosynthesis: importance of thylakoid reactions, rubisco deactivation, reactive oxygen species, and thermotolerance provided by isoprene. *Plant Cell Environ.* 28 269–277. 10.1111/j.1365-3040.2005.01324.x

[B33] Shi W. Muthurajan R. Rahman H. Selvam J. Peng S. Zou Y. (2013). Source-sink dynamics and proteomic reprogramming under elevated night temperature and their impact on rice yield and grain quality. *New Phytol.* 197 825–837. 10.1111/nph.12088 23252708

[B34] Sinsawat V. Leipner J. Stamp P. Fracheboud Y. (2004). Effect of heat stress on the photosynthetic apparatus in maize (*Zea mays* L.) grown at control or high temperature. *Environ. Exp. Bot.* 52 123–129. 10.1016/j.envexpbot.2004.01.010

[B35] Sui N. Li M. Liu X. Wang N. Fang W. Meng Q. (2007). Response of xanthophyll cycle and chloroplastic antioxidant enzymes to chilling stress in tomato over-expressing glycerol-3-phosphate acyltransferase gene. *Photosynthetica* 45 447–454. 10.1007/s11099-007-0074-5

[B36] Talukder A. S. Mcdonald G. K. Gill G. S. (2013). Effect of short-term heat stress prior to flowering and at early grain set on the utilization of water-soluble carbohydrate by wheat genotypes. *Field Crops Res.* 147 1–11. 10.1016/j.fcr.2013.03.013

[B37] Talukder H. Mcdonald G. Gill G. (2014). Effect of short-term heat stress prior to flowering and early grain set on the grain yield of wheat. *Field Crops Res.* 160 54–63. 10.1016/j.fcr.2014.01.013

[B38] Tian Y. Chen J. Chen C. Deng A. Song Z. Zheng C. (2012). Warming impacts on winter wheat phenophase and grain yield under field conditions in Yangtze Delta Plain, China. *Field Crops Res.* 134 193–199. 10.1016/j.fcr.2012.05.013

[B39] Wahid A. Gelani S. Ashraf M. Foolad M. R. (2007). Heat tolerance in plants: an overview. *Environ. Exp. Bot.* 61 199–223. 10.1016/j.envexpbot.2007.05.011

[B40] Walter J. Nagy L. Hein R. Rascher U. Beierkuhnlein C. Willner E. (2011). Do plants remember drought? Hints towards a drought-memory in grasses. *Environ. Exp. Bot.* 71 34–40. 10.1016/j.envexpbot.2010.10.020

[B41] Wang X. Cai J. Jiang D. Liu F. Dai T. Cao W. (2011). Pre-anthesis high-temperature acclimation alleviates damage to the flag leaf caused by post-anthesis heat stress in wheat. *J. Plant Physiol.* 168 585–593. 10.1016/j.jplph.2010.09.016 21247658

[B42] Wang X. Cai J. Liu F. Dai T. Cao W. Wollenweber B. (2014). Multiple heat priming enhances thermo-tolerance to a later high temperature stress *via* improving subcellular antioxidant activities in wheat seedlings. *Plant Physiol. Biochem.* 74 185–192. 10.1016/j.plaphy.2013.11.014 24308988

[B43] Wang X. Cai J. Liu F. Jin M. Yu H. Jiang D. (2012). Pre-anthesis high temperature acclimation alleviates the negative effects of post-anthesis heat stress on stem stored carbohydrates remobilization and grain starch accumulation in wheat. *J. Cereal Sci.* 55 331–336. 10.1016/j.jcs.2012.01.004

[B44] Xu S. Li J. Zhang X. Wei H. Cui L. (2006). Effects of heat acclimation pretreatment on changes of membrane lipid peroxidation, antioxidant metabolites, and ultrastructure of chloroplasts in two cool-season turfgrass species under heat stress. *Environ. Exp. Bot.* 56 274–285. 10.1016/j.envexpbot.2005.03.002

[B45] Yang Z. Yin F. Wang X. Li Y. Ren J. Yin J. (2014). Regulating effect of accumulated temperature prior to wintering of light use efficiency in winter wheat (*Triticum aestivum* L.). *J. Nucl. Agric. Sci.* 28 1489–1496.

[B46] Yucel M. Burke J. J. Nguyen H. T. (1992). Inhibition and recovery of photosystem II following exposure of wheat to heat shock. *Environ. Exp. Bot.* 32 133–135. 10.1016/0098-8472(92)90037-3

[B47] Zadoks J. C. Chang T. T. Konzak C. F. (1974). A decimal code for the growth stages of cereals. *Weed Res.* 14 415–421. 10.1111/j.1365-3180.1974.tb01084.x

[B48] Zahoor R. Dong H. Abid M. Zhao W. Wang Y. Zhou Z. (2017). Potassium fertilizer improves drought stress alleviation potential in cotton by enhancing photosynthesis and carbohydrate metabolism. *Environ. Exp. Bot.* 137 73–83. 10.1016/j.envexpbot.2017.02.002

[B49] Zhang X. Zhou Q. Wang X. Cai J. Dai T. Cao W. (2016). Physiological and transcriptional analyses of induced post-anthesis thermo-tolerance by heat-shock pretreatment on germinating seeds of winter wheat. *Environ. Exp. Bot.* 131 181–189. 10.1016/j.envexpbot.2016.08.002

[B50] Zhao H. Dai T. Jiang D. Cao W. (2008). Effects of high temperature on key enzymes involved in starch and protein formation in grains of two wheat cultivars. *J. Agron. Crop Sci.* 194 47–54. 10.1111/j.1439-037X.2007.00283.x

[B51] Zheng C. Dong J. Liu F. Dai T. Qi J. Cao W. (2009). Effects of salt and waterlogging stresses and their combination on leaf photosynthesis, chloroplast ATP synthesis, and antioxidant capacity in wheat. *Plant Sci.* 176 575–582. 10.1016/j.plantsci.2009.01.015 26493148

